# Bacteriophage K1F targets *Escherichia coli K1* in cerebral endothelial cells and influences the barrier function

**DOI:** 10.1038/s41598-020-65867-4

**Published:** 2020-06-01

**Authors:** Christian Møller-Olsen, Toby Ross, Keith N. Leppard, Veronica Foisor, Corinne Smith, Dimitris K. Grammatopoulos, Antonia P. Sagona

**Affiliations:** 1 School of Life Sciences, University of Warwick, Gibbet Hill Road, CV4 7AL Coventry, UK; 2 Warwick Medical School, University of Warwick, Gibbet Hill Road, CV4 7AL Coventry, UK; 3 Department of Chemistry, University of Warwick, Gibbet Hill Road, CV4 7AL Coventry, UK; 4 Institute of Precision Diagnostics and Translational Medicine, Dept of Pathology, UHCW NHS Trust, Clifford Bridge Road, CV2 2DX Coventry, UK; 5 Warwick Integrative Synthetic Biology Centre, University of Warwick, Coventry, CV47AL UK

**Keywords:** Cell biology, Microbiology

## Abstract

Bacterial neonatal meningitis results in high mortality and morbidity rates for those affected. Although improvements in diagnosis and treatment have led to a decline in mortality rates, morbidity rates have remained relatively unchanged. Bacterial resistance to antibiotics in this clinical setting further underlines the need for developing other technologies, such as phage therapy. We exploited an *in vitro* phage therapy model for studying bacterial neonatal meningitis based on *Escherichia coli* (*E. coli*) EV36, bacteriophage (phage) K1F and human cerebral microvascular endothelial cells (hCMECs). We show that phage K1F is phagocytosed and degraded by constitutive- and PAMP-dependent LC3-assisted phagocytosis and does not induce expression of inflammatory cytokines TNFα, IL-6, IL-8 or IFNβ. Additionally, we observed that phage K1F temporarily decreases the barrier resistance of hCMEC cultures, a property that influences the barrier permeability, which could facilitate the transition of immune cells across the endothelial vessel *in vivo*. Collectively, we demonstrate that phage K1F can infect intracellular *E. coli* EV36 within hCMECs without themselves eliciting an inflammatory or defensive response. This study illustrates the potential of phage therapy targeting infections such as bacterial neonatal meningitis and is an important step for the continued development of phage therapy targeting antibiotic-resistant bacterial infections generally.

## Introduction

Bacterial meningitis is characterised by severe inflammation of the meninges, a network of connective tissues surrounding the brain and spinal cord. The inflammation is a result of bacterial invasion into the subarachnoid space, between the arachnoid and pia mater and can be caused by numerous bacterial species with meningococci, pneumococci, Group B streptococci and *Escherichia coli* being the most prevailing species^1,2^. These acute and potentially life-threatening infections require rapid diagnosis and initiation of treatment, usually administration of antibiotics and sometimes corticosteroids. The disease is more common in neonates and young children as their immune systems are relatively immature, with incidence rates of 1/5,000 in full-term neonates and 1/500 in low-birth-weight neonates in developed countries. The mortality rate for treated bacterial neonatal meningitis is reported at 5–20%, with significant life-changing neurological sequelae for 25–50% of survivors that include cognitive impairment, deafness, blindness and seizures^3,4^. *E. coli* strains are a prevalent cause of bacterial neonatal meningitis^5^, in particular, strains that express the K1 capsule^6^, an α-2,8-linked polysialic acid polymer, that covers the surface of the bacteria thus hiding many of its antigenic features. This capsule is believed to enhance its ability to evade the human immune system and to traverse the blood-brain-barrier (BBB)^5,6^, highlighting the clinical importance of this particular pathogen as a therapeutic target.

Improvements in diagnosis and treatment of bacterial neonatal meningitis have seen a gradual decline in mortality rates in recent decades, while long term post-infection morbidity rates have remained relatively unchanged^3^. However, the emergence of antibiotic resistance is a major cause of concern and could lead to a resurgence in mortality rates; this is supported by recent epidemiological studies showing declining antibiotic susceptibility of clinical isolates derived from the cerebrospinal fluid of meningitis patients^7^. As an increasing number of infections are becoming harder or impossible to treat, there is an urgent need for the development of technologies that can complement or replace conventional antibiotics.

As a re-emerging technology, phage therapy holds great potential for the treatment of resistant and non-resistant bacterial infections, and promising results have been achieved in cases of compassionate use^8^. Virulent bacteriophages (or phages) propagate within a suitable host at the site of infection, ultimately lysing that host and repeating the cycle, allowing for the potential of single-dose administration to eradicate a large number of bacterial cells^9^. Phage therapy was reported to successfully treat meningitis caused by multiple pathogens as early as at the turn of the 21^st^ century^10,11^ manifesting the ability of phages to cross physiological barriers, including the BBB^12^, whereas many antimicrobials, including vancomycin, beta-lactams and other hydrophilic antibiotics have reduced penetration across the BBB^13^. Studies performed in a rat meningitis model infected with *E. coli* O25b:H4-ST131, a strain producing extended-spectrum beta-lactamase CTx-M-15, showed 100% and 50% rat survival following administration of phage EC200^PP^ 7 or 24 h post-infection respectively^14^.

A comprehensive understanding of phage interactions with human cells on a cellular and molecular level is desired to be able to accurately substantiate the efficacy of the approach. Phages are present on all body surfaces that are in direct contact with the exterior environment, including the skin, urogenital tract, oral cavity, gut and lungs^15^, in addition to the blood^16^. While their presence in body niches allows them to exert selective pressure on their bacterial hosts and hence to modulate the human microbiome, their presence in the blood allows for direct interaction with mammalian immune cells and the potential for induction of innate and adaptive immune responses^17^. Furthermore, the presence of phages in the blood might allow for direct contact with vascular endothelial cells with unknown biological consequences, yet the potential influence and significance of such interactions are yet to be explored.

We present a robust *in vitro* phage therapy model system of neonatal bacterial meningitis based on *E. coli* EV36, phage K1F and hCMECs allowing for a wide range of cellular and molecular analyses. We show that, in hCMEC cultures, phage K1F is phagocytosed and degraded by constitutive- and inducible PAMP-dependent LC3-assisted phagocytosis, does not initiate selective autophagy, and does not induce expression of inflammatory cytokines TNFα, IL-6, IL-8 or IFNβ. Conversely, *E. coli* EV36 is degraded by selective autophagy, and a potent inflammatory response is mounted towards the infection in the form of expression of inflammatory cytokines. Additionally, we show that phage K1F temporarily decrease the impedance of hCMEC cultures. This decrease in focal adhesion influences the endothelial barrier function by increasing the barrier permeability and this might represent a mechanism allowing for the transition of immune cells across the endothelial vessel.

## Results

### *E. coli* EV36 infects human cerebral microvascular endothelial cells in culture

**Figure 1:**
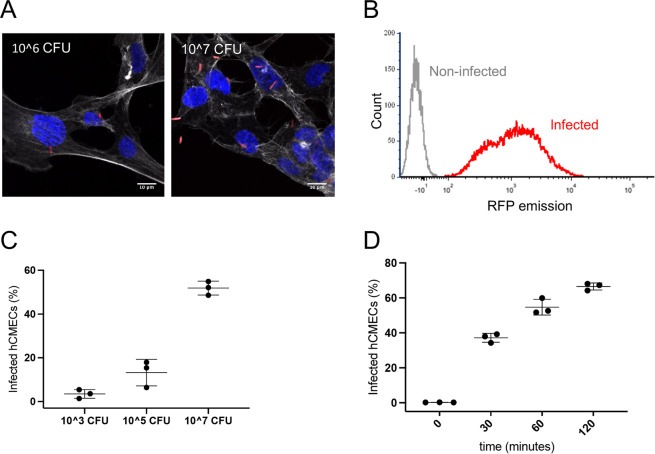
Infection by *E. coli* EV36-RFP of hCMEC cultures. (**A**) Representative fluorescent images showing *E. coli* EV36-RFP infection of hCMEC cultures at concentrations of 10^6^ CFU/ml (**left**) and 10^7^ CFU/ml (**right**). *E. coli* EV36-RFP fluorescence shown in red, DAPI in blue, and phalloidin in white (**B**) Flow cytometry histogram showing *E. coli* EV36-RFP infected hCMECs in red and non-infected hCMECs in grey, as detected by RFP fluorescence. (**C,D**) Mean percentages of *E. coli* EV36-RFP infected hCMECs (RFP-positive by flow cytometry) after a 1 h incubation period with concentrations of 10^3^ to 10^7^ CFU/ml (**C**), or 10^7^ CFU/ml sampled over time (**D**); + /− SD, n = 3 in each case.

Infection rates of hCMECs by *E. coli* EV36-RFP were determined by confocal microscopy and flow cytometry (Fig. 1).

The proportion of hCMECs containing intracellular bacteria, as quantified by confocal imaging, was increased in a concentration-dependent manner (Fig. 1A & Suppl. Figure 1). Estimates of infection rates in larger cell populations were obtained by complementary approaches using flow cytometry (Fig. 1B–D). Full separation of RFP fluorescence signals from infected and non-infected cultures was achieved (Fig. 1B). The proportion of infected hCMECs increased in a concentration-dependent manner with increasing bacterial input (Fig. 1C). Additionally, a clear time-dependence of infection was observed in cultures infected at a single concentration (Fig. 1D).

The comparable results obtained by confocal imaging and flow cytometry demonstrated that both methods are applicable for quantifying *E. coli* infection of cultured hCMEC and that this infection is both concentration- and time-dependent.

### Bacteriophage K1F host specificity

**Figure 2:**
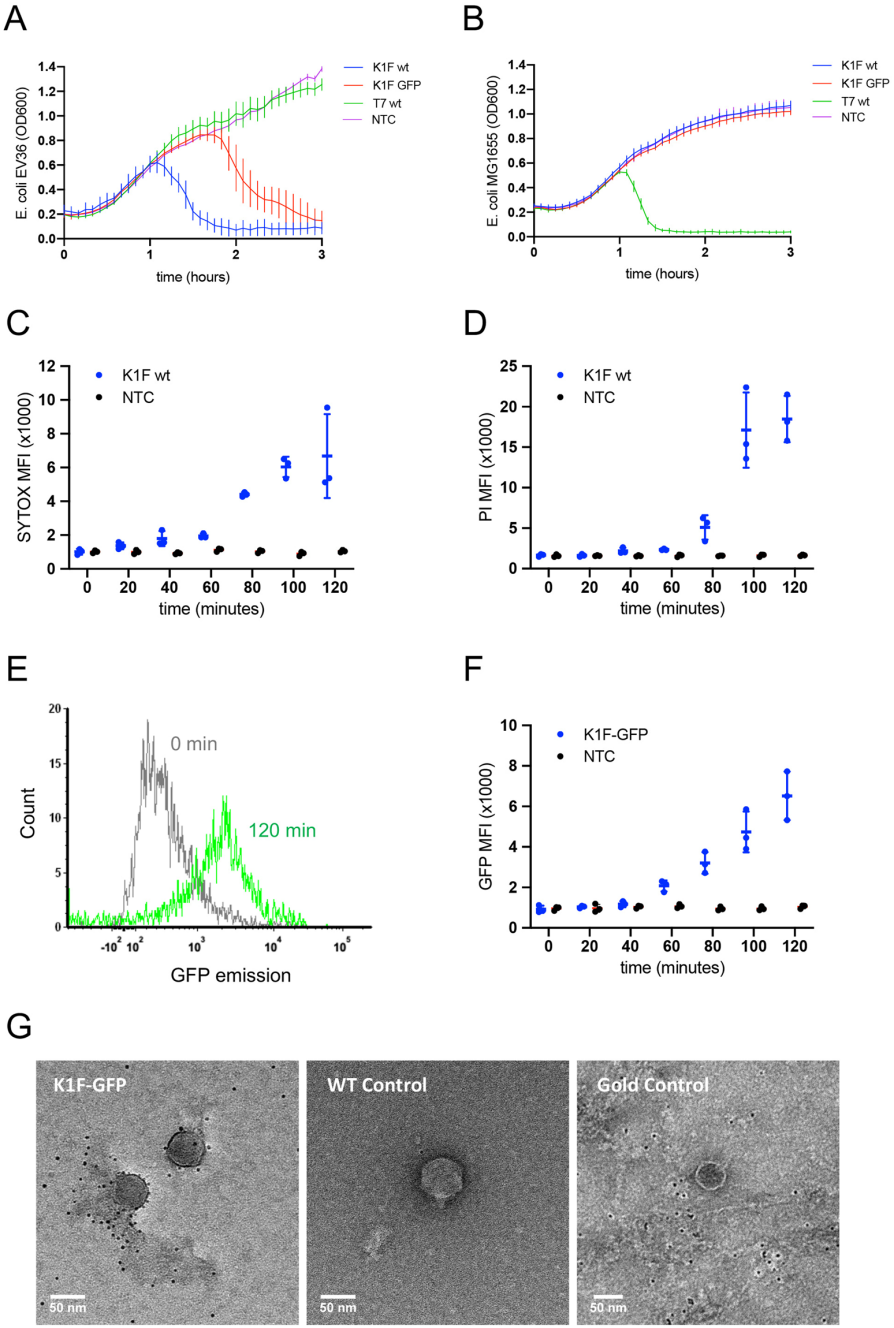
Bacteriophage K1F infection of *E. coli* EV36. (**A,B**) Growth of *E. coli* EV36 (**A**) and *E. coli* MG1655 (**B**) cultures infected by phages K1F, K1F-GFP or T7 at a MOI of 0.001 in comparison with uninfected control cultures, NTC (+/− SD, n > 3). (**C–E**) Analysis of bacterial cell death in phage-infected cultures by flow cytometry; *E. coli* EV36 cultures were infected with phage at a MOI of 0.001; NTC = uninfected control (**C**) Sytox Green Dead Cell Stain MFI (+/− SD, n = 3) of phage K1F infected *E. coli* EV36. (**D**) Propidium iodide (PI) MFI (+/− SD, n = 3) of phage K1F infected *E. coli* EV36. (**E**) Flow cytometry histogram of phage K1F-GFP infected *E. coli* EV36 at 0 min following infection (grey) or 120 min (green). (**F**) GFP MFI (+/− SD, n = 3) of phage K1F-GFP infected *E. coli* EV36; NTC = uninfected Control. (**G**) Negative staining EM images of sample (Gold-conjugated GFP binding probe with phage K1F-GFP) and controls. Wild-type control is represented by phage K1F incubated with the probe; Gold control was performed using only the 5 nm gold incubated with phage K1F-GFP.

The host specificity of phages K1F and K1F-GFP towards K1 capsule-expressing bacteria was confirmed by liquid culture growth assays (Fig. 2A,B).

The addition of phages caused the optical density of *E. coli* EV36 cultures to plateau and then to decrease, indicating successful infection (Fig. 2A), with phage K1F-GFP showing some delay in infection in comparison with phage K1F, whereas growth continued unhindered after addition of phage T7. In contrast, phages K1F and K1F-GFP were unable to infect *E. coli* MG1655 as no decrease in optical density was observed (Fig. 2B) whereas phage T7 successfully infected this strain. These data confirmed the high specificity of phages K1F and K1F-GFP towards *E. coli* strains such as EV36, which express the K1 capsule, and also suggest that GFP expression by phage K1F-GFP has some small fitness cost as it kills its host more slowly than phage K1F.

### Effect of bacteriophage K1F on the viability and cell wall integrity of E. coli EV36

The effect of phage K1F infection on the viability and cell wall integrity of *E. coli* EV36 (Fig. 2C,D) was investigated using flow cytometry.

The addition of phage K1F decreased viability and cell wall integrity of *E. coli* EV36 cultures over time as indicated by an increasing mean fluorescence intensity (MFI) of the membrane-impermeant nucleic acid dyes Sytox (Fig. 2C) and propidium iodide (PI) (Fig. 2D). The temporal differences observed between the two dyes are likely due to the properties and intensities of the dyes themselves. It is noteworthy that a decreasing rate of events was detected from 80 minutes onwards due to bacterial lysis, and that no events were detectable after 140 minutes, suggesting that no live bacteria were present.

### Stability of phage K1F-GFP

The stability of phage K1F-GFP was further investigated using flow cytometry (Fig. 2E,F) and electron microscopy (EM) (Fig. 2G).

Increasing GFP fluorescence was observed of *E. coli* EV36 cultures infected with phage K1F-GFP (Fig. 2F) over time. No events were recorded after 140 minutes incubation due to bacterial lysis. Two distinguishable populations were observed when comparing the GFP-tagged phage at 0 h and 2 h (Fig. 2E).

Additionally, EM imaging showed that the gold-conjugated probe for GFP binds to phage K1F-GFP in a unique pattern (Fig. 2G); the phages are decorated with the probe in a ring-like shape surrounding the capsid, suggesting that there is enhanced binding of the probe to phage K1F-GFP in comparison with the controls, which showed no specific binding of the probe. This data confirmed the presence and high stability of the GFP fluorophore located on the capsid of phage K1F-GFP.

### Endotoxin concentration of phage preparations

The concentration of endotoxin, LPS, in phage preparations used to treat human cell cultures was determined using a Limulus Amebocyte Lysate assay. This was performed to ensure that the molecular and cellular changes observed was caused by phage interactions rather than bacterial remains. Supplementary Fig. 4 shows that phage added to cultures at 10^7^ PFU/ml contained 0.0060–0.086 EU/ml and cultures with phage added at 10^9^ PFU/ml contained 0.60–1.3 EU/ml. Endotoxin concentrations in this range would enable intravenous administration of phage at a high titre while being well below the recommended 5 EU per kg of body weight per hour^18^. While the experimental concentration of endotoxins in this study is very low, the potential effect of trace bacterial contaminants is not accounted for. Therefore, interpretation of data could be skewed by currently unknown effects of trace bacterial debris or interactions of these with phage particles.

### Internalisation and degradation of bacteria and phages in human cerebral microvascular endothelial cells

The internalisation and degradation of phage K1F-GFP and *E. coli* EV36 in T24 human urinary epithelial cells has previously been studied in the context of *in vitro* phage therapy^19^. Here we expanded on this study using qualitative confocal microscopy and quantitative flow cytometry to examine the internalization and processing of phage K1F in human cerebral microvascular endothelial cells (hCMECs).

**Figure 3:**
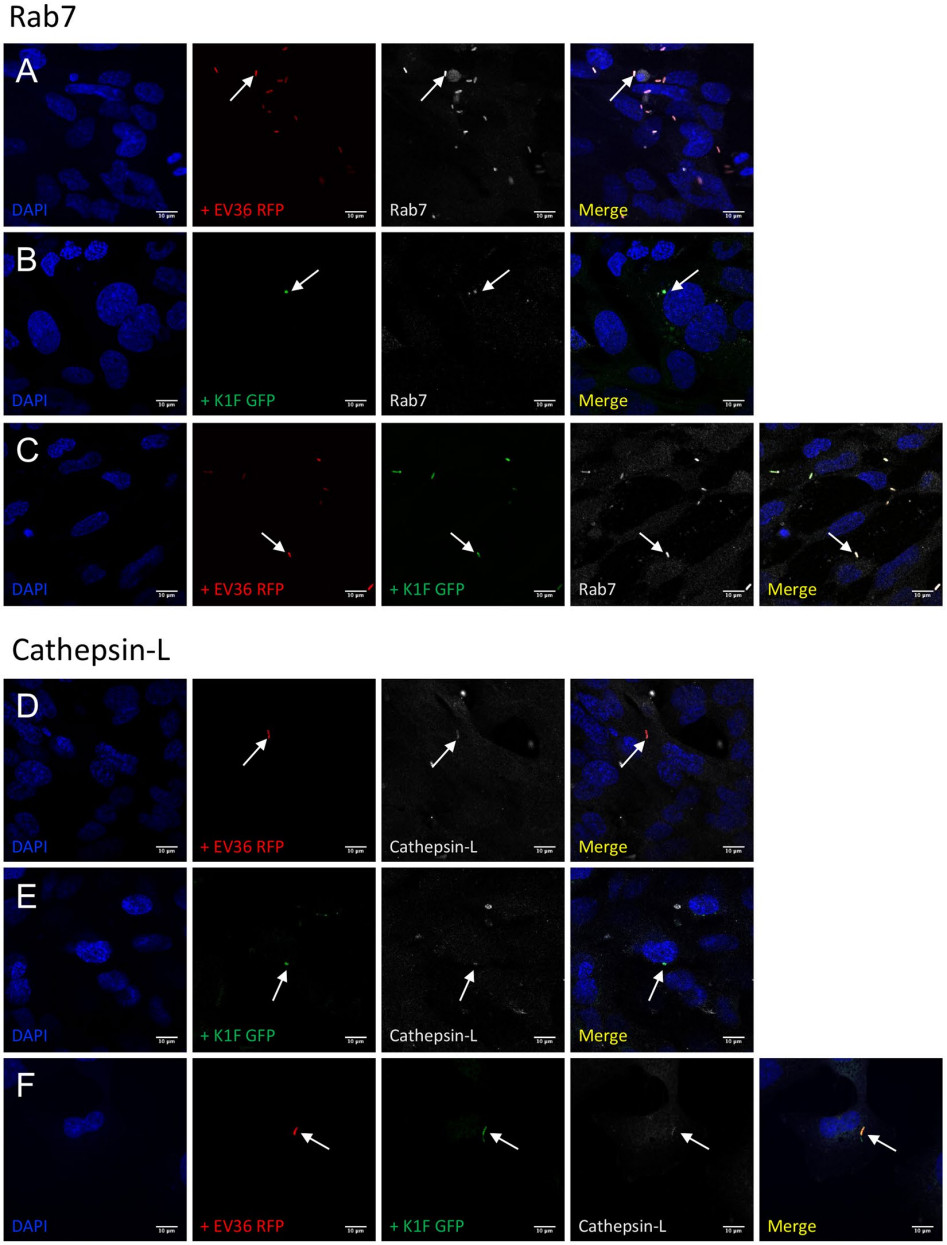
Lysosomal- and phagosomal markers of constitutive phagocytosis. (**A–F**) Immunofluorescent images showing hCMEC cultures fixed and stained with anti-RAB7 (**A–C**) and anti-Cathepsin-L (**D–F**) antibodies following a 1 h incubation with 10^7^ CFU/ml *E. coli* EV36-RFP alone (**A** + **D**) or 10^7^ PFU/ml phage K1F-GFP alone (**B** + **E**), or a 1 h incubation with 10^7^ CFU/ml *E. coli* EV36-RFP followed by a 1 h incubation with 10^4^ PFU/ml phage K1F-GFP (**C** + **F**). DAPI stain is shown in blue and anti-RAB7/anti-Cathepsin-L antibodies in white. n = 3 in each case.

**Figure 4:**
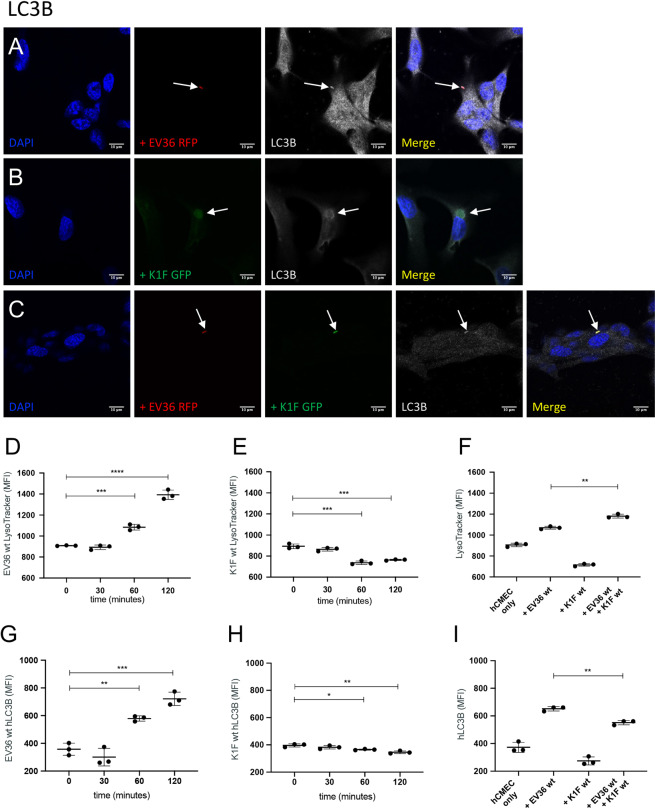
Markers of LC3-assisted phagocytosis and lysosomal activity. (**A–C**) Immunofluorescent images showing hCMEC cultures fixed and stained with anti-LC3B antibody following a 1 h incubation with 10^7^ CFU/ml *E. coli* EV36-RFP alone (**A**) or 10^7^ PFU/ml phage K1F-GFP alone (**B**), or a 1 h incubation with 10^7^ CFU/ml *E. coli* EV36-RFP followed by a 1 h incubation with 10^4^ PFU/ml phage K1F-GFP (**C**). DAPI stain is shown in blue and anti-LC3B antibody in white. n = 3 in each case. (**D,E**) Analysis by flow cytometry. (**D–F**) Graphs showing LysoTracker MFI (+/− SD, n = 3) of hCMEC cultures incubated with 10^7^ CFU/ml *E. coli* EV36 alone (**D**) or 10^7^ PFU/ml phage K1F alone (**E**) over time, or in combination after 1 h incubation (**F**). (**G,H**) Graphs showing anti-hLC3B MFI (+/− SD, n = 3) of hCMEC cultures treated with 10^7^ CFU/ml *E. coli* EV36 (**G**) or 10^7^ PFU/ml phage K1F (**H**) over time, or in combination after 1 h incubation (**I**). Probability values (p-values) are displayed as p ≤ 0.05 (*), p ≤ 0.01 (**), p ≤ 0.001 (***), p ≤ 0.0001 (****).

Assessment of internalisation and degradation was initially performed as a co-localisation assay between bacteria or phage and phagosomal and lysosomal markers. hCMEC cultures were fixed and stained with antibodies for either RAB7, a GTPase required for the normal progression of late endosomes to lysosomal fusion^20,21^ (Fig. 3A–C), cathepsin-L, a cysteine protease important for lysosomal degradation of engulfed extracellular material^22^ (Fig. 3D–F), or LC3B, a microtubule-associated protein that is recruited by toll-like receptors for LC3-associated phagocytosis and autophagosome formation^23^ (Fig. 4A–C).

Co-localisation assays in hCMEC cultures showed that RAB7, cathepsin-L, and LC3B, all co-localise with *E. coli* EV36-RFP (Fig. 3A,D & 4A), phage K1F-GFP (Fig. 3B,E & 4B), or *E. coli* EV36-RFP and phage K1F-GFP in combination (Fig. 3C,F & 4C). RAB7 and cathepsin-L co-localisation suggested that *E. coli* EV36-RFP and phage K1F-GFP alike are internalised by constitutive phagocytosis and following a maturation process, these vesicles fuse with lysosomes. Constitutive phagocytosis is a continual process whereby the human cells sample the extracellular space and uptake inert particles such as some nutrients^24^. Additionally, LC3B co-localisation suggests that components of the autophagy pathway are activated via inducible LC3-assisted phagocytosis^25^.

Confocal microscopy image analysis also confirmed observations of previous studies performed in T24 human urinary epithelial cells^19^, showing the ability of phage K1F-GFP to locate and infect *E. coli* EV36-RFP in a human cell environment and the formation of intracellular vacuoles containing phage-aggregates in human cells.

In addition, quantitative flow cytometry was used to measure the MFI of LysoTracker (Fig. 4D–F) and hLC3B (Fig. 4G,H). The LysoTracker is a pH-sensitive, membrane-permeable dye that labels acidic organelles with high selectivity^26^. The data showed a sharp increase over time of LysoTracker MFI of hCMEC cultures treated with *E. coli* (Fig. 4D), suggesting a shift in endosomal pathway activity towards an increased lysosomal activity, whilst hCMEC cultures treated with phage K1F showed a lesser gradual decrease (Fig. 4E). hCMEC cultures treated with *E. coli* EV36 and phage K1F in combination showed a small increase of LysoTracker MFI in comparison with cells treated with *E. coli* EV36 alone (Fig. 4F). This increased LysoTracker activity observed, is likely the result of an increase in bacterial debris, in the form of lipopolysaccharides (LPS), released by phage lysis.

Similarly, staining of hCMEC cultures stained with an hLC3B antibody showed a sharp increase over time following incubation with *E. coli* EV36 (Fig. 4G), while hCMEC cultures treated with phage K1F showed a lesser gradual decrease (Fig. 4H). hCMEC cultures treated with *E. coli* EV36 and phage K1F in combination (Fig. 4I) showed a small but significant decrease of hLC3B MFI in comparison with *E. coli* EV36 treatment alone. This observed decrease could translate to a quicker progression from phagosome maturation to lysosome degradation induced by the release of endotoxins following phage lysis.

### Degradation of bacteria and phages by xenophagy

**Figure 5:**
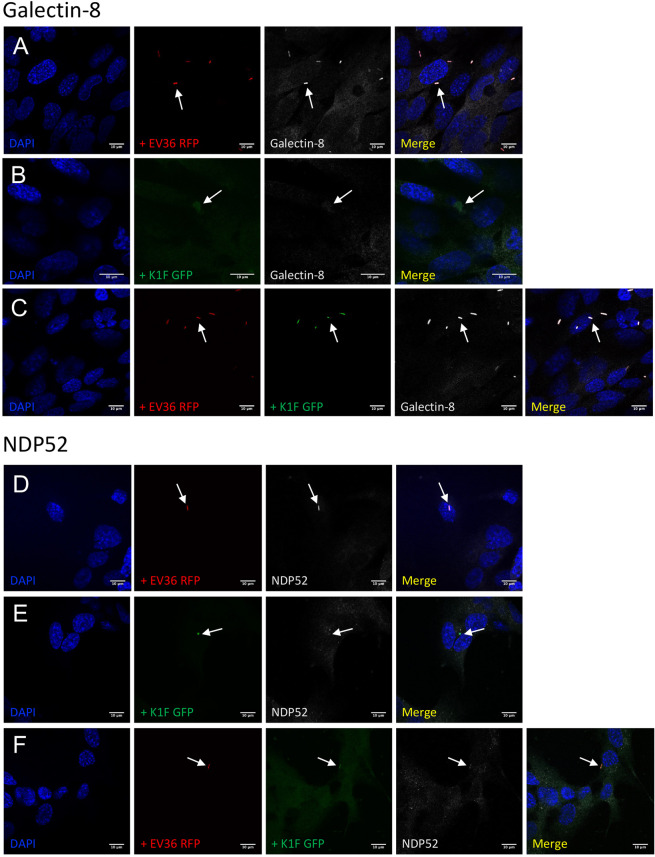
Markers of xenophagy. (**A–F**) Immunofluorescent images showing hCMEC cultures fixed and stained with anti-Galectin-8 (**A–C**) and anti-NDP52/CALCOCO2 (**D–F**) antibodies following a 1 h incubation with 10^7^ CFU/ml *E. coli* EV36-RFP alone (**A** + **D**) or 10^7^ PFU/ml phage K1F-GFP alone (**B** + **E**), or a 1 h incubation with 10^7^ CFU/ml *E. coli* EV36-RFP followed by a 1 h incubation with 10^4^ PFU/ml phage K1F-GFP (**C** + **F**). DAPI stain is shown in blue and anti-Galectin-8/anti-NDP52 antibodies in white. n = 3 in each case.

**Figure 6:**
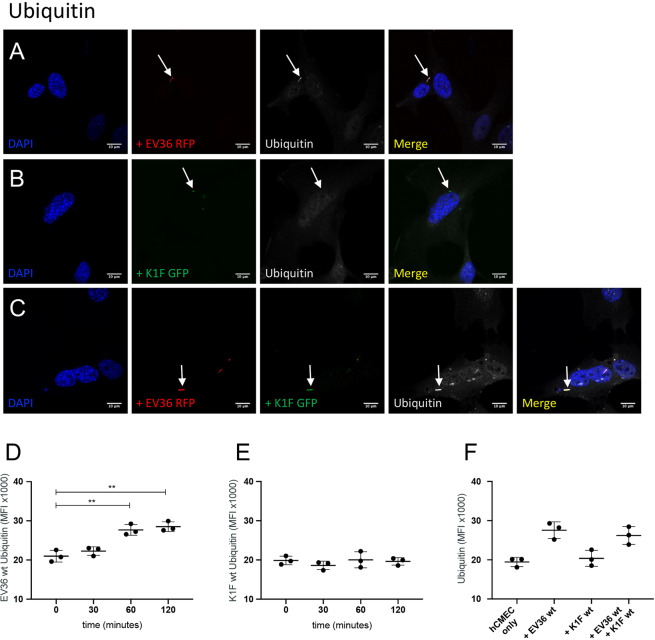
Ubiquitin targeted degradation. (**A–C**) Immunofluorescent images showing hCMEC cultures fixed and stained with anti-Ubiquitin antibody following a 1 h incubation with 10^7^ CFU/ml *E. coli* EV36-RFP alone (**A**) or 10^7^ PFU/ml phage K1F-GFP alone (**B**), or a 1 h incubation with 10^7^ CFU/ml *E. coli* EV36-RFP followed by a 1 h incubation with 10^4^ PFU/ml phage K1F-GFP (**C**). DAPI stain is shown in blue and anti-Ubiquitin antibody in white. n = 3 in each case. (**D–F**) Graphs showing anti-Ubiquitin MFI (+/− SD, n = 3) obtained by flow cytometry of hCMEC cultures incubated with 10^7^ CFU/ml *E. coli* EV36 alone (**D**) or 10^7^ PFU/ml phage K1F alone (**E**) over time, or in combination after 1 h incubation (**F**). Probability values (p-values) are displayed as p ≤ 0.05 (*), p ≤ 0.01 (**), p ≤ 0.001 (***), p ≤ 0.0001 (****).

Alongside LC3-assisted phagocytosis, anti-bacterial selective autophagy (xenophagy) can occur when bacteria, internalised by phagocytosis, attempt to escape into the cytosol by rupturing the encapsulating membrane^25^. A further three antibodies were selected to distinguish between LC3-assisted phagocytosis and xenophagy: Galectin-8, a cytosolic lectin which monitors endosomal membrane integrity and responds to membrane damage by binding to exposed glycans^27^ (Fig. 5A–C), NDP52, a cargo-receptor that, when recruited by Galectin-8, initiates the formation of an autophagosome which triggers selective autophagy^28^ (Fig. 5D–F) and ubiquitin, the deposition of which on exposed cytosolic bacteria is sensed by a range of receptors, including NDP52, and ultimately restricting bacterial proliferation and initiating xenophagy^29^ (Fig. 6A–C).

Intracellular *E. coli* EV36-RFP in hCMEC cultures co-localised with antibodies for Galectin-8 (Fig. 5A), NDP52 (Fig. 5D) and ubiquitin (Fig. 6A), suggesting that *E. coli* EV36-RFP is capable of rupturing the phagosomal membrane and initiating xenophagy. In contrast, image data showed no co-localisation between intracellular vacuoles containing phage K1F-GFP and either Galectin-8 (Fig. 5B), NDP52 (Fig. 5E), or ubiquitin (Fig. 6B), suggesting that phage K1F-GFP is solely degraded by constitutive- and LC3-assisted phagocytosis and does not initiate xenophagy. Additionally, hCMEC cultures infected with *E. coli* EV36-RFP and phage K1F-GFP in combination displayed identical co-localisation patterns to that of *E. coli* EV36-RFP infection alone, with co-localisation of both bacterial and phage signals with antibodies for Galectin-8 (Fig. 5C), NDP52 (Fig. 5F) and ubiquitin (Fig. 6C), indicative of xenophagy.

Additionally, flow cytometry was performed to quantify the ubiquitin-mediated contribution to degradation (Fig. 6D–F). hCMEC cultures incubated with *E. coli* EV36 showed an increase in ubiquitin MFI over time eventually reaching a plateau (Fig. 6D), while no difference was observed of hCMEC cultures incubated with phage K1F alone (Fig. 6E) or *E. coli* EV36 and phage K1F in combination (Fig. 6F). These data replicated the results obtained by imaging, suggesting that *E. coli* EV36 alone and not phage K1F, can initiate xenophagy.

### Expression pattern of inflammatory markers of hCMEC cultures

**Figure 7:**
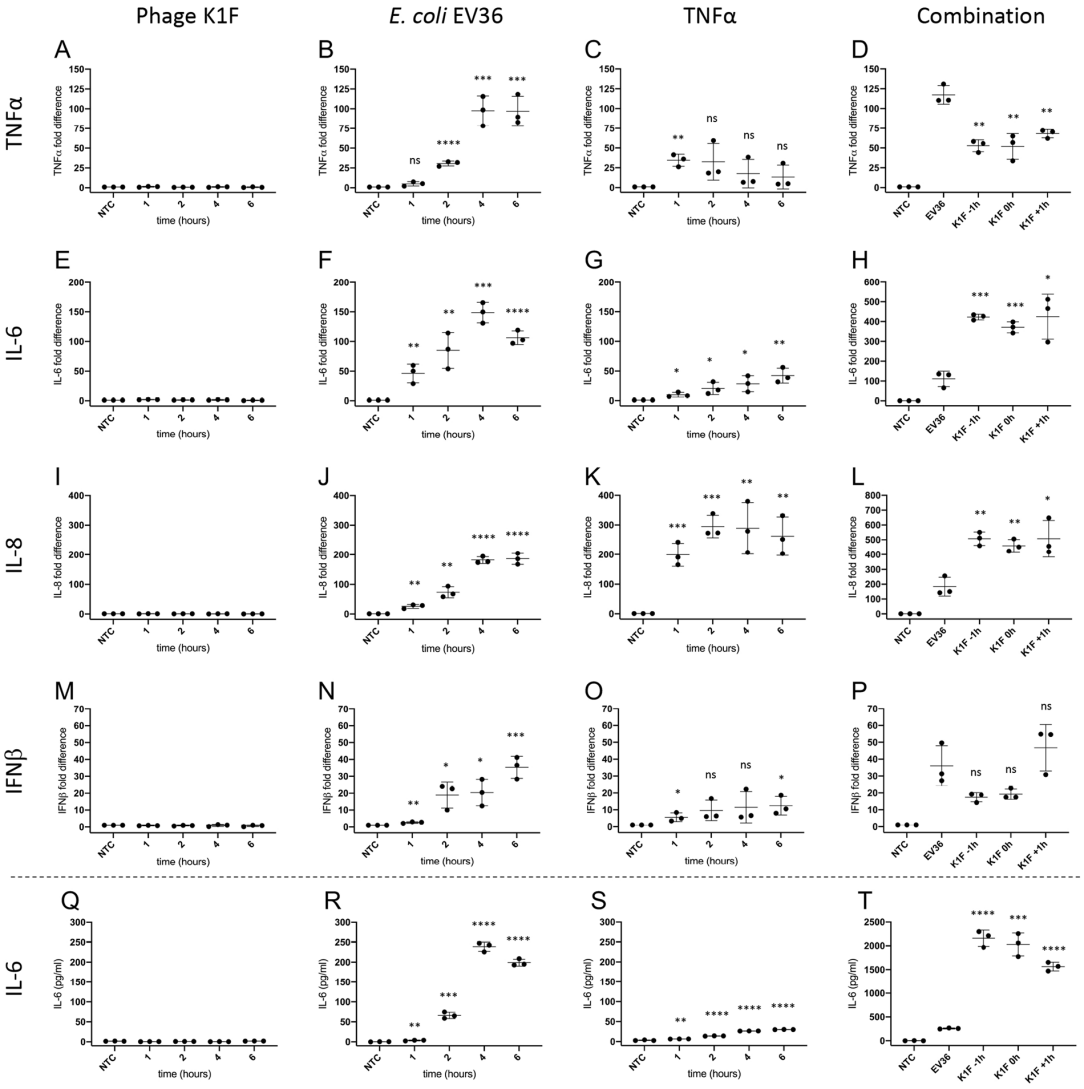
Expression pattern of inflammatory markers by real-time qPCR (**A–P**). hCMEC cultures were incubated with 10^7^ CFU/ml *E. coli* EV36, 10^7^ PFU/ml phage K1F or 500 pg/ml TNFα and specific mRNA levels measured over time, or incubated with 10^7^ CFU/ml *E. coli* EV36 having 10^4^ PFU/ml phage K1F added 1 h before-, simultaneously-, or 1 h after bacterial addition, and incubated for 6 h before RNA harvest. Real-time qPCR was performed with primer pairs specific for TNFα (**A–D**), IL-6 (**E–H**), IL-8 (**I–L**) and IFNβ (**M–P**). Data were expressed relative to internal control (GAPDH) and then normalised to the untreated control value. Expression of IL-6 protein as measured by ELISA (**Q-T**). hCMEC cultures were incubated with 10^7^ PFU/ml phage K1F, 10^7^ CFU/ml *E. coli* EV36 or 500 pg/ml TNFα, or incubated with 10^7^ CFU/ml *E. coli* EV36 having 10^4^ PFU/ml phage K1F added 1 h before-, simultaneously-, or 1 h after bacterial addition, and incubated for 6 h. NTC = Untreated cultures. +/− SD, n = 3 in each case. Probability values (p-values) are displayed as p ≤ 0.05 (*), p ≤ 0.01 (**), p ≤ 0.001 (***), p ≤ 0.0001 (****) and not statistically significant p ≥ 0.05 (ns). P-values are relative to NTC for single treatments experiments and relative to *E. coli* EV36 for combination treatment experiments.

The inflammatory response of human cerebral endothelial cells to *E. coli* EV36 and phage K1F individually and in combination was investigated by measuring the induction of inflammatory markers TNFα, IL-6, IL-8, IL-10 and IFNβ using real-time qPCR. hCMEC cultures treated with TNFα was included as a positive control for induction of expression (Fig. 7). TNFα is considered a master regulator of inflammation and is implicated in the pathogenesis of bacterial, viral, and chronic inflammatory disease. In response to TNFα, vascular endothelial cells will increase leucocyte adhesion and trans-endothelial migration illustrating its central role as a pro-inflammatory cytokine in clearing an infection^30^. While hCMEC cultures treated with phage K1F showed no change in the expression levels of TNFα over time (Fig. 7A), cultures treated with *E. coli* EV36 showed a significant and sharp increase (Fig. 7B). The TNFα expression induced by *E. coli* EV36 infection was reduced by approximately 50% with the addition of phage K1F (Fig. 7D). The onset of measurable TNFα induction occurs from 2 hours incubation with bacteria and beyond (Fig. 7B), which correlated with bacterial clearance (Fig. 2), suggesting that the measured expression at the 6-hour endpoint is independent of the presence of live bacteria.

During infections, IL-6, a pleiotropic cytokine involved in both acute and acquired immunity, and also in neural development and function, is rapidly produced to stimulate host defences such as induction of C-reactive protein expression, the concentration of which is directly related to the severity of infection^31–33^. Over time, hCMECs treated with phage K1F showed no statistically significant change in the expression levels of IL-6 (Fig. 7E), while cultures treated with *E. coli* EV36 (Fig. 7F) showed a gradual time-dependent increase. Control cultures treated with TNFα (Fig. 7G) showed a weaker response characterised by a gradual time-dependent increase throughout the incubation period. Contrary to TNFα, IL-6 expression induced by *E. coli* EV36 treatment (Fig. 7H) was increased more than 200% with the addition of phage K1F, whilst no difference was observed between phage K1F incubation time points. This suggests that, in the context of phage therapy, the inflammation measured as the expression of IL-6 of hCMEC cultures is mainly induced by the influx of bacterial endotoxins released by phage lysis rather than the bacterial infection itself. TNFα and IL-6 are typically both induced by such a stimulus, often attributed to the action of NF-κB; however, the TNFα response to NF-κB is complex and negatively affected by the action of other cell type-specific factors that compete for its binding to the promoter^34^ which may explain the discordance in activation of TNFα and IL-6 in our experiments.

IL-8 is a potent chemokine involved in the inflammatory response, specifically attracting and activating neutrophils at the site of infection, and is associated with many chronic inflammatory conditions^35^. hCMEC cultures incubated with phage K1F showed no difference in IL-8 expression over time (Fig. 7I). *E. coli* EV36 treatment, on the contrary, induced a time-dependent increase of IL-8 expression of hCMEC cultures (Fig. 7J). A faster onset of increased IL-8 expression was observed in hCMEC cultures treated with TNFα (Fig. 7K). The induced expression of IL-8 following *in vitro* phage therapy of hCMEC cultures (Fig. 7L), was comparable to that of IL-6 expression, showing an average increase above 150% following phage K1F addition in relation to *E. coli* EV36 treated cultures alone. No difference was observed between the three time points of phage K1F addition. As with IL-6 induction, the data suggests that the hCMECs respond in a higher degree to bacterial endotoxins released by bacterial clearance following phage therapy that the actual bacterial infection itself.

The production of inflammatory cytokines in response to a stimulus is mediated mainly through new transcription of cytokine genes^36^ and indeed levels of cytokine mRNA were close to zero in the absence of a stimulus. We confirmed mRNA induction by stimulus was reflected in protein levels for IL-6 as an exemplar, using ELISA (Fig. 7Q–T). For all conditions, there was a striking correlation between IL-6 protein levels over time and their respective mRNA expression profiles.

The anti-inflammatory cytokine, IL-10, is known to play a role in limiting host immune responses towards pathogens^37^. In these experiments, the data showed no change in IL-10 expression over time following incubation with *E. coli* EV36 or phage K1F alone or in combination (Suppl. Figure 5).

IFNβ was included in these experiments to complement the broadly inflammatory cytokines already measured with a cytokine that has a known virus inducible expression. Expression of IFNs, including IFNβ, has been shown to be induced by activation of pattern recognition receptors in the early stages of viral infection^38,39^.

hCMEC cultures incubated with phage K1F showed no induced expression of IFNβ over time (Fig. 7M), however, cultures incubated with *E. coli* EV36 showed a small time-dependent increase of IFNβ expression (Fig. 7N). hCMEC cultures incubated with TNFα showed a barely significant induction of IFNβ (Fig. 7O). hCMEC cultures treated with both *E. coli* EV36 and phage K1F exhibited responses characterised by a greater variation within and between experiments of induced expression of IFNβ yielding no statistically significant differences between treatments. Collectively, the IFNβ data suggest that phage K1F does not induce inflammatory responses of IFNβ in hCMEC cultures and that the IFNβ expression induced by *E. coli* EV36 is more variable and smaller than the induced expression of TNFα, IL-6 and IL-8.

### Temporal impedance profiles of hCMEC cultures

**Figure 8:**
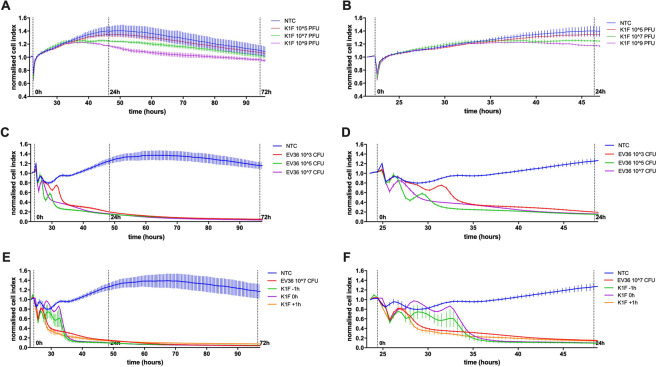
Temporal impedance profiling as measured using the xCELLigence system (**A–F**). Graphs displaying the barrier resistance over time of hCMEC cultures treated with phage K1F at a concentration range of 10^5^ to 10^9^ PFU/ml (**A,B**), *E. coli* EV36 at a concentration range of 10^3^ to 10^7^ CFU/ml (**C,D**), or combination treatment of 10^7^ CFU/ml *E. coli* and 10^4^ PFU/ml phage K1F added 1 h before-, simultaneously-, or 1 h after bacterial addition (**E,F**). NTC = Untreated hCMEC cultures. X-axis show time from cell seeding. Vertical lines denote the addition of treatment at 0 h, and 24- and 72 h post treatment. The data is presented as the average normalised cell index across the acute (24 hours) and long-term (72 hours) incubate period (+/− SD, n > 3).

The temporal influence of *E. coli* EV36 and phage K1F alone and in combination on the barrier function of hCMEC cultures was determined using the xCELLigence system (Fig. 8). The temporal impedance profile of hCMEC cultures treated with phage K1F showed a slow divergence between NTC and phage K1F treated cultures at the highest concentrations of 10^7^ and 10^9^ PFU with detectable signal after approx. 12 hours incubation (Fig. 8B). The decrease in impedance due to phage K1F was sustained long-term (Fig. 8A), and reached maximum levels after approx. 35 hours incubation; a reduced response was detected by around 72-hour of incubation. To assess if these observations were specific to phage K1F, a similar experiment was performed using phage T7. This experiment showed a similar concentration-dependent decrease (Suppl. Figure 2), suggesting that these observations could be relevant to responses to phage in general. Additionally, as the impedance measurement is affected not only by cell-to-electrode adhesion but also cell proliferation, a control experiment was performed measuring the proliferation of hCMEC cultures treated with phage K1F or T7 at identical concentrations. This experiment showed no difference in cell proliferation over time between NTC and cultures treated with phages (Suppl. Figure 3), suggesting that the proliferation of hCMEC cultures is not influenced by phage addition.

The impedance of hCMEC cultures treated with *E. coli* EV36 showed an immediate and dramatic decrease over the initial 10 hours for all concentrations (Fig. 8D). The detrimental effect of *E. coli* EV36 infection on the hCMEC cultures is clear with no recovery during the experiment (Fig. 8C), ending at a decrease of >95% across all bacterial concentrations.

In modelling several *in vitro* phage therapy intervention protocols, phage K1F was added to hCMEC cultures 1 hour before, simultaneously, and 1 hour after the addition of *E. coli* EV36 (Fig. 8E,F). The immediate and dramatic influence of bacterial infection is similar to experiments without phage addition, however, the addition of phage K1F 1 hour prior to or simultaneously with *E. coli* EV36 addition, allowed the impedance of hCMEC cultures to transiently recover (Fig. 8F) in the initial hours of infection. Ultimately, however, despite phage intervention, the impedance was not recovered beyond the initial 10 hours of incubation and similar effects with a decrease of >95% across all treatments were observed.

## Discussion

In this study, we have aimed to develop an *in vitro* phage therapy model for studying bacterial neonatal meningitis. As part of this process, we initially infected hCMEC cultures with *E. coli* EV36. We present that the infection rate of hCMEC cultures is time and concentration-dependent. The percentage of individual hCMECs containing intracellular bacteria was quantified by microscopy and flow cytometry. The two methods of quantification yielded very similar time and concentration-dependent results validating their application. hCMEC cultures incubated with 10^7^ CFU *E. coli* EV36-RFP for 1 h showed infection rates of approx. 50%. This is considerably higher than the infection rate previously obtained of approx. 20% in human urinary bladder epithelial cells (T24)^19^. This highlighted inherent tissue-specific properties and the necessity for unique tissue-specific model systems to study the mechanism of bacterial infection and phage intervention. We further tested the bacteriophage K1F which we found to display high host specificity towards *E. coli* EV36. The specificity of phage K1F against its bacterial host *E. coli* EV36 was confirmed in liquid cultures, where phages K1F and K1F-GFP were shown to lyse *E. coli* EV36 cultures and not *E. coli* MG1655. Conversely, Phage T7 was shown to lyse *E. coli* MG1655 and not *E. coli* EV36. This demonstrates the high host specificity of the phages used in this study. Infection dynamics assessed by flow cytometry in the form of bacterial viability and cell wall integrity by Sytox^40^ and Propidium Iodide (PI)^41^ staining respectively, showed a population Mean Fluorescence Intensity (MFI) increase of over time of both Sytox and PI culminating immediately before bacterial clearance. It is notable that both stains are membrane-impermeable and thus the fluorescent signal is only present in the brief interval from initial lysing to complete dismantlement of the bacterial membrane.

The ability and efficiency of phage K1F-GFP to locate and infect intracellular *E. coli* EV36 in a human cell environment has been confirmed in previous studies^19^, however, the signal stability of GFP is often a point for discussion. We therefore initially assessed the fluorescent signal emitted by phage K1F-GFP during infection in *E. coli* EV36 using flow cytometry. We show that bacterial cultures infected with phage K1F-GFP for two hours have a distinctly different fluorescent profile than non-infected bacterial cultures. The fluorescence emitted was time-dependent with a continual increase until bacterial clearance. The location of individual GFP molecules was confirmed by electron microscopy, showing the unequivocal presence of individual GFP molecules across the capsid in alignment with the engineering design of bacteriophage K1F-GFP which places it on the minor capsid protein. These results are in accordance with a previous study^42^, which demonstrated that the gp10b minor capsid protein is present in a limited number of copies, around 15 copies per capsid, in comparison with the major gp10a protein, which forms most of the capsid and is expressed in around 429 copies.

In support of previous results obtained in urinary epithelial cells (T24)^19^, our initial imaging data show that *E. coli* EV36 and phage K1F individually and in combination co-localise with antibodies for RAB7, Cathepsin-L and LC3B in hCMEC cultures. The protein targets of these antibodies are involved in phagosome maturation^20,21^, lysosomal degradation^22^ and autophagosome formation^23^ respectively. Collectively, this illustrates a path of internalisation and degradation of phage K1F by constitutive phagocytosis succeeded by lysosomal degradation along the pathway of LC3-assisted phagocytosis.

LC3-assisted phagocytosis is dependent on the recognition of Pathogen Associated Molecular Patterns (PAMPs) by Toll-like Receptors (TLRs)^43^, suggesting that mammalian cells recognise phages via TLRs in order to degrade via them via this pathway. Multiple intracellular TLRs are able to recognise viral nucleic acids, specifically, TLR3 recognises dsRNA, TLR7 and TLR8 recognises ssRNA and TLR9 recognises DNA^44^.

The concentration of acidic organelles, including lysosomes, as measured by LysoTracker, decreases over time following phage K1F invasion in hCMEC cultures. It is feasible that the accessible pool of lysosomes is being exhausted during phage degradation, while the cells are not immediately replenishing this store. This is seen in sharp contrast to the substantial increase of lysosome concentration following *E. coli* EV36 infection in hCMEC cultures. Interestingly, phage addition to *E. coli* EV36 infected hCMECs increases the lysosomal activation additionally. This is likely linked to the increase in bacterial debris following bacterial lysis, as the presence of LPS from gram-negative bacteria is a known inducer of autophagy^45^ leading to increased lysosome activity.

While we show that phage K1F and *E. coli* EV36 are degraded by constitutive and LC3-assisted phagocytosis in hCMEC cultures, it is evident that *E. coli* strains can transit from the phagosome into the cytosol in an attempt to evade the innate immune response of lysosomal degradation^25,46^. In line with previous observations in T24 cells^19^, our imaging efforts show that *E. coli* EV36 and not phage K1F co-localise with antibodies for Galectin-8, NDP52 and Ubiquitin in hCMEC cultures. These antibodies target proteins involved in the initiation- and progression of xenophagy, which is pathogen-selective autophagy^25,47^. Specifically, breaches to the phagosomal membrane are sensed by cytosolic Galectin-8 which binds to exposed membrane-bound glycans^27^ and interacts with cytosolic NDP52 to mediate autophagosome formation^28^ thus triggering selective autophagy. Ubiquitination of the escaped bacterium^47^ and the Galectin-8-NDP52 complex^25^ further promote pathogen degradation. This finding is substantiated by quantitative flow cytometry data showing an increase of ubiquitin abundance over time in hCMEC cultures infected with *E. coli* EV36, whereas hCMEC cultures invaded with phage K1F show no difference of ubiquitin abundance over time.

Real-time qPCR analysis demonstrated that phage K1F does not induce the expression of inflammatory cytokines TNFα, IL-6, IL-8 or IFNβ by hCMEC cultures. In contrast, phage upregulation of TNFα and IL-6 expression has been observed *in vitro* in peripheral blood monocytes (PBMCs)^48^. The inflammatory response of PBMCs conforms to the nature of this collection of immune cells, whereas the absence of an inflammatory response of hCMEC cultures is compatible with the nature and function of the endothelium^49^ despite their known involvement in immune processes^50^. Expression of Type I interferons, including IFNβ, can be induced by nucleic acid-recognising TLRs^44^ and PAMPs such as LPS. While this study found phages were degraded, in part, by PAMP-recognition dependent LC3-assisted phagocytosis, no increase was observed in IFNβ expression of hCMEC cultures following phage invasion. Further studies would be required to investigate which TLR(s) is associated with phage degradation via the LC3-assisted phagocytic pathway. The absence of induction of inflammatory cytokines by phage K1F is seen in sharp contrast to the substantial upregulation of expression induced by *E. coli* EV36 in particular TNFα, IL-6 and IL-8. This is in line with previously published bacterial infection patterns of the human endothelium^51^. Interestingly, *in vitro* phage therapy using bacteriophage K1F against *E. coli* EV36 infection of hCMEC cultures resulted in a reduction in TNFα expression of approximately 50% compared to bacterial infection alone. As a central pro-inflammatory cytokine in bacterial infection^30^, the reduction observed in TNFα expression is likely related directly to the reduction in bacterial concentration as a result of phage intervention.

Conversely, a steep increase was observed following *in vitro* phage therapy in the expression of IL-6 and IL-8. The outcome of phage intervention is bacterial lysis and a high concentration of bacterial debris, LPS. This increase in cytokine expression correlates well with results showing LPS induction of IL-6 and IL-8 expression in lymphatic microvascular cells (LECs)^52^.

Finally, we present that bacteriophage K1F decreases the barrier function of hCMEC cultures.

hCMEC cultures incubated with phage K1F at concentrations of approximately 10^7^ PFU/ml and above showed a temporal reduction of impedance over a considerable length of time.

As far as we are aware this is the first time this has been reported. Similar temporal profiles were observed for incubation with phage T7. Further control experiments showed that these observations were unrelated to hCMEC proliferation.

The frequency of 10 kHz used in these experiments principally favours the measurement of cellular focal adhesion^53^. The cellular change indicated by a decrease in impedance is weaker focal adhesion to the extracellular matrix, suggesting that the endothelial barrier function is decreased and thus the endothelium is becoming more permeable in the presence of high concentrations of phage. Circulating free phage is rapidly degraded once the host bacteria have been cleared in *in vivo*^9,14^ and *in vitro*^54^ tests of phage intervention, which suggests that the recovery of hCMEC impedance observed here following prolonged incubation was directly linked to a reduction in bacteriophage concentration. *E. coli* EV36 infection in hCMEC cultures resulted in a rapid and total reduction in barrier resistance in line with previous *in vivo*^55^ and *in vitro*^56^ studies of endothelial barrier function during infection. Interestingly, phage intervention only conferred a short-lived recovery of barrier resistance, ultimately leaving the hCMEC culture to perish due to increasing concentration of LPS.

Continual stimulation of antiviral immune responses by commensal bacteriophage activation of innate immune pathways has been shown to induce low-level cytokine production, resulting in continuously activated immune cells that confer protection against pathogenic viral infections^17,57^. Additionally, a non-host-derived layer of immunity is theorised to result from phages residing in mucosal layers (BAM model), whereby the presence of phages at the site-of-entry for pathogenic bacteria confers immunity as well as impacting the mucosal-resident community of commensal bacteria^58^. We hypothesise that modulation of endothelial adhesion could be added to the list as an inflammatory mechanism supporting the delivery and extravasation of immune cells to a site of infection.

Overall, the model we propose here to study the interaction between K1F phage, its host bacteria and hCMEC human cells, even though it is in initial steps in terms of directly approaching a clinical phage therapy intervention, can give us useful information to be used when phage therapy is considered. Based on our results, K1F phage is safe to be used as it does not increase inflammation in hCMEC cells and targets successfully its host *Escherichia coli K1*. More studies are required to provide further proof that the current model can be linked even more accurately with real phage therapy examples of neonatal meningitis.

## Conclusion

Collectively, the presented results expand the growing understanding of interactions between phages and human cells, reinforce the existing knowledge of phage internalisation and degradation, complemented by an analysis of expression patterns of inflammatory cytokines during *in vitro* phage therapy and results showing the temporal influence of phages on the barrier permeability of human endothelial cells. While there is evidence of interactions between bacteriophage and human cells, it is clear that the responses mounted by the human cells are not inflammatory or defensive. The contribution of this study is valuable in the continued development of phage therapy for bacterial neonatal meningitis and the ongoing war against antibiotic-resistant bacterial infections.

## Materials and Methods

### Human cell culture

The blood-brain barrier hCMEC/D3^59^ (human cerebral microvascular endothelial cells) cell line (Merck, UK) consists of enriched cerebral microvascular endothelial cells immortalised by lentiviral vector transduction with the catalytic subunit of human telomerase (hTERT) and SV40 large T antigen.

hCMECs were cultured in EndoGRO-MV Complete Media (Merck) supplemented with 1 ng/ml bFGF (FGF-2) (Merck), 100 IU/ml Penicillin (Sigma-Aldrich), and 100 μg/ml Streptomycin (Sigma-Aldrich) and maintained under a humidified atmosphere at 37 C in 5% CO_2_. All culture vessels were coated with 5 μg/cm^2^ Collagen Type 1 (Collagen-1) (Merck) in PBS for 1 h at 37 C before use.

### Preparation of hCMEC cultures for experiments

For immunocytochemical (ICC) imaging, flow cytometry and real-time qPCR hCMECs were seeded onto 6-well plates in culture medium and allowed 48 h to settle. The seeding density for ICC imaging was approx. 2.1 × 10^4^ cells/cm^2^, and approx. 5.2 × 10^4^ cells/cm^2^ for flow cytometry and real-time qPCR.

Prior to an experiment, the culture medium was replaced with Leibovitz L-15 media (Lonza), a medium designed to support cell growth in environments lacking CO_2_ equilibration, and the cultures were moved to a 37 C incubator suitable for bacterial infections.

### Growth determination of hCMEC cultures

hCMEC cells were seeded in culture medium onto 96-well tissue culture plates at a density of approx. 1.5 × 10^4^ cells/cm^2^ and allowed 24 hours to settle. Phages T7 or K1F were added to corresponding wells with untreated cells included as a control. The cell density was determined at the time of seeding and at subsequent sampling points by manually using a haemocytometer on a trypsinised population.

### Bacterial culture

Three bacterial strains were used in this study: 1) *E. coli* EV36, a K12/K1 hybrid derivative^60^, was kindly provided by Drs Eric R. Vimr and Susan M. Steenbergen. This hybrid strain allowed for work to be performed in a biohazard level 1 laboratory while retaining the phenotypic properties of a pathogenic K1 strain. 2) *E. coli* EV36-RFP, an EV36 derivative constructed by electroporation with low-copy plasmid pSB6A1 constitutively expressing the mRFP1 protein and cultured under 100 μg/ml ampicillin selection. 3) *E. coli* MG1655 (ATCC 47076) (LGC Standards, UK), a well-documented K12 strain, that was included as a control for phage selectivity.

### Bacteriophage propagation and purification

Three phage strains were used in this study: 1) Phage K1F, a well-characterised strain that shows high specificity towards K1-capsule expressing bacteria^61^, was kindly provided by Dr Dean Scholl. The K1 polysaccharide capsule of *E. coli* is recognised and degraded by a phage K1F-encoded endosialidase allowing phage infection^6^. 2) Phage K1F-GFP, a phage K1F derivative engineered by genome integration of GFP as previously described^19^. 3) Phage T7^62^, an extensively used strain showing high specificity towards commensal *E. coli* K12 strains.

Single-clone phage preparations were obtained from cleared bacterial cultures following a modified Castro-Mejia *et al*.^63^ protocol previously published^19^. Briefly, this comprised the release of phage particles from bacterial membranes by NaCl addition, phage precipitation by PEG8000 addition, followed by CsCl gradient column separation, and size exclusion by dialysis. The concentration of endotoxins (LPS) in purified phage preparations were determined using the Limulus Amebocyte Lysate (LAL) Chromogenic Endpoint Assay (Hycult Biotech) following the manufacturer’s protocol. Endotoxin concentrations in phage preparations, media and diluents used in this study are presented in Suppl. Figure 4.

Phage host selectivity was assessed by phage infection of liquid bacterial cultures. Phages K1F, K1F-GFP or T7 were added to *E. coli* EV36 or *E. coli* MG1655 in a 96-well plate and the optical density at 600 nm was measured using FLUOstar Omega (BMG Labtech). Uninfected cultures and LB alone were included as controls.

### Flow cytometry analysis of phage infection in bacteria

Log-phase *E. coli* EV36 cultures were stained with 2 μM final concentration propidium iodide (PI) (Invitrogen) or 3 μM final concentration Sytox Green Dead Cell Stain (Invitrogen), and incubated for 20 min before the addition of phage K1F.

Assessment of fluorophore stability was performed by the addition of phage K1F-GFP alone to a separate bacterial culture.

Data acquisition and analysis were performed using the LSR Fortessa flow cytometer and FACSDiva software (BD Biosciences). Bacterial populations were initially gated on size via SSC/FCA to exclude cellular debris. 10,000 events were detected of each population. The mean fluorescent intensity (MFI) was detected for gated bacterial populations using B488-530/30 A optics for Sytox Green and phage K1F-GFP detection, and YG561-586/15 for propidium iodide detection.

### Electron microscopy of phage K1F-GFP

The probe was generated using 5 nm gold functionalized with maleimide groups (Sigma-Aldrich) conjugated to a GFP binding nanobody which has been proven to bind with a high efficiency to GFP proteins^64^, while similar approaches of nanobodies conjugated to gold have shown to be able to track GFP proteins in cells^65^. This approach allows tracking of the GFP protein present on the gp10b capsid protein, as the nanobody binds to the GFP and the gold allows visualization with EM. For the binding of the probe to phage K1F-GFP, 2 µL of 10^8^ PFU/ml phage was incubated with 1 µL of the probe (1.8 × 10^−6^ mol/L concentration) at room temperature for 1 hr. Following this, 5 µL of 1% PBS was added and the total volume was incubated on a formvar/carbon-coated grid (EMResolutions) that was previously glow discharged for 1 min. After 10 minutes, the grid was washed 3 times with 1% PBS, followed by 4 minutes incubation with 2% uranyl acetate staining. The grid was imaged using a Jeol 2100Plus TEM microscope fitted with a Gatan OneView IS camera at 200 kV. Gold controls and wild-type controls were generated using the same protocol, with phage K1F-GFP incubated with only 5 nm gold functionalized with maleimide groups or phage K1F incubated with the probe, respectively. The acquired images were then processed using Fiji (imageJ) software.

### Immunofluorescent confocal microscopy

hCMEC cultures were fixed in 4% paraformaldehyde (ThermoFisher Scientific) for 15 min, permeabilised in ice-cold PEM buffer (80 mM PIPES pH 6.75, 5 mM EGTA, 1 mM MgCl_2_) with 0.05% (v/v) Saponin for 5 min, and quenched with 50 mM NH_4_Cl in PBS for 15 min. PBS washes were performed after each step.

For association assays, the fixed cells were stained with the following primary antibodies diluted in 0.05% Saponin in PBS for 60 min at room temperature, as described also previously^19^: 40 μg/ml anti-RAB7 (Bioss Inc, MA); 1 μg/ml anti-Cathepsin L (Abcam); 5 μg/ml anti-LC3B (Sigma-Aldrich); 1 μg/ml anti-CALCOCO2/NDP52 (Abcam); 1 μg/ml anti-Galectin-8 (R&D Systems); or 1 μg/ml anti-mono- and polyubiquitinylated conjugated monoclonal antibody (FK2) (Enzo Life Sciences). This was followed by detection with secondary antibodies by incubation for 45 min at room temperature with Cy5 Affinipure Donkey Anti-goat, Anti-rabbit or Anti-mouse IgG (Jackson ImmunoResearch, PA). The signal from GFP tagged phage was further enhanced with 5 μg/ml GFP-Booster (Chromotek, Germany) during incubation with secondary antibodies. For invasion assays, the fixed cells were stained only with 5 μg/ml Phalloidin CF680R Conjugate (Biotium) for 40 min.

Stained coverslips were mounted on microscope slides using DAPI-containing Fluoroshield Mounting Medium (Abcam, UK) and secured with CoverGrip Coverslip Sealent (Biotium). Finally, the cells were imaged using a Zeiss LSM880 confocal microscope with Airyscan, using the following excitation wavelengths: DAPI at 405 nm, GFP at 561 nm, RFP at 561 nm and far-red (Cy5) at 633 nm.

### Flow cytometry analysis of infection in human cells

Sampling was performed by aspirating spent media, trypsinising and centrifuging each the suspension. MEDIUM A (Fix & Perm Cell Permeabilization Kit) (Life Technologies) was added to the pellet and incubated according to the manufacturer’s instructions. The cell suspension was diluted in eBioscience Flow Cytometry Staining Buffer (Invitrogen) and pelleted by centrifugation. MEDIUM B (Fix & Perm Cell Permeabilization Kit) (Life Technologies) containing either 1/100 dilution anti-hLC3B Alexa Fluor 647 Conjugated Ab (R&D Systems), or 1 ug/ml Anti-mono- and polyubiquitinylated conjugated monoclonal antibody (FK2) (Enzo Life Sciences), was added to the sample and incubated for 45 minutes. Following incubation, the cell suspension was diluted in Staining Buffer and pelleted by centrifugation as above. For ubiquitin staining only, a secondary incubation with MEDIUM B containing 2 ug/ml Goat anti-mouse IgG (H + L) Cross Absorbed Secondary Ab Alexa Fluor 647 (Invitrogen) was performed for 30 minutes. Finally, the cell suspension was resuspended in Staining Buffer and placed on ice until acquisition.

MEDIUM A and MEDIUM B were not supplemented with antibodies for LysoTracker (Invitrogen, UK) staining. LysoTracker was added to samples 30 minutes prior to trypsinisation at a final concentration of 500 nM. The endocytic pathway follows a pH gradient ranging from ~6.3 in early endosomes, ~5.5 in late endosomes, to ~4.6 in lysosomes^66^. An observed increase of LysoTracker MFI indicates a downstream shift in endosomal pathway activity towards increased lysosomal activity.

hCMECs treated with *E. coli* EV36-RFP alone were analysed both fixed and live. For fixed cells, the Fix & Perm Cell Permeabilization Kit was used as above. For live cells, the trypsinised cells were diluted in PBS and Sphero Beads AccuCount Fluorescent Particles (SpheroTech, IL) were added according to the manufacturer’s instructions, and data acquired immediately thereafter.

Acquisition and analysis were performed using the LSR Fortessa flow cytometer and FACSDiva software (BD Biosciences). 10,000 events were detected of each population. The hCMEC populations were initially gated on size via SSC/FCA, excluding cellular debris and planktonic bacteria. The mean fluorescent intensity (MFI) was detected for gated hCMEC populations using the following optics: R640-670/14-A for LysoTracker and Alexa Fluor 647 conjugated Abs, and YG561-586/15 for *E. coli* EV36-RFP.

### Quantitative real-time PCR

hCMEC cultures were incubated with *E. coli* EV36, phage K1F or TNFα (Sino Biological, China) in biological triplicates. RNA was recovered using the GenElute Mammalian Total RNA Miniprep Kit (Sigma-Aldrich) with DNase I Digestion Kit (Sigma-Aldrich) following the manufacturer’s protocol.

1 μg RNA samples were used for cDNA synthesis using 100 U Superscript III (Invitrogen) primed by 50 nM Random Hexamers (Invitrogen) and 10 μM dNTP Mix (Thermo Scientific).

Real-time PCR of cDNA samples was performed with Brilliant III SYBR Green qPCR Master Mix with Low ROX (Agilent) using the Stratagene Mx3005P instrument with MxPro v4.10 build 389 software (Agilent Technologies). Each sample was quantified in technical triplicate using primer sets for GAPDH^67^, TNFα^68^, IL-6^69^, IL-8^70^, IL-10^71^ and IFNβ^72^.

Data analysis was performed using the Comparative Ct Method^73^. ΔCt values for TNFα, IL-6, IL-8, IL-10 and IFNβ were calculated by subtracting the average GAPDH Ct value from the average Ct value obtained for each gene. ΔΔCt values were calculated by subtracting ΔCt values of untreated cultures from ΔCt values of treated cultures. The fold change was calculated by taking the log base 2 of ΔΔCt values.

### Detection of IL-6 protein expression using ELISA

hCMEC cultures were incubated with *E. coli* EV36, phage K1F or TNFα (Sino Biological, China) in biological triplicates. Spent media was recovered, centrifuged, sterile filtered and stored at −80C until analysis. Expression of IL-6 protein was detected using the Human IL-6 Uncoated ELISA kit (Invitrogen) following the manufacturer’s instructions.

### Temporal impedance measurements of human cells

hCMECs were seeded into Collagen-1 coated E-Plate VIEW 16 PET (ACEA Biosciences) at a concentration of 5 × 10^4^ cells/well in assay media. The plates were then inserted into the RTCA DP station and the cells allowed approx. 24 h to settle before the addition of bacteria or phage. The acquisition was performed using the xCELLigence RTCA DP instrument (ACEA Biosciences) housed in a humidified incubator at 37 C with 5% CO_2_. The instrument was set to a single frequency of 10 kHz with 5 min measuring intervals over the course of 96 h. Using a fixed frequency of 10 kHz, the current predominantly travels paracellularly allowing for impedance measurements relating to the cell-to-electrode adhesion (focal adhesion) of the cells^53^. The parameter measured by the xCELLigence system, the Cell Index, represents the relative changes in frequency-dependent electrode resistance or impedance. Changes observed in impedance of confluent endothelial cells reflect changes in barrier function^74^. The Cell Index was normalised to 1 relative to approx. 1 h before the addition of treatments.

### Quantification and statistical analysis

All quantification and statistical analysis were performed using GraphPad Prism 8.2.1 (San Diego, CA). Probability values for flow cytometry- and qPCR datasets were calculated using the unpaired t-test assuming Gaussian distribution. Level of significance is presented in relevant graphs.

## Supplementary information

Supplemental information.
