# Supplementary material for: Bacteriophage K1F targets Escherichia coli K1 in cerebral endothelial cells and influences the barrier function
Source: Sci Rep. 2020 Jun 1;10:8903. doi: 10.1038/s41598-020-65867-4 (PMC7264188; doi:10.1038/s41598-020-65867-4)
Supplement: Supplementary file 1 — Supplemental information. [file 41598_2020_65867_MOESM1_ESM.pdf]

Supplementary material

Title

Bacteriophage K1F influences the barrier function of cerebral endothelial cells in an *in vitro* phage therapy model of neonatal meningitis.

Authors

Christian Møller-Olsen [1] ORCID: 0000-0002-6944-5601
Toby Ross [1] ORCID: 0000-0001-7277-6949
Keith N. Leppard [1] ORCID: 0000-0003-2155-7919
Veronica Foisor [3] ORCID: 0000-0001-7004-1324
Corinne Smith [1] ORCID: 0000-0003-3364-7946
Dimitris K. Grammatopoulos [2, 4] ORCID: 0000-0002-6296-8290
Antonia P. Sagona [1] * ORCID: 0000-0002-0386-2322

Affiliations:

[1] School of Life Sciences, University of Warwick, Gibbet Hill Road, CV4 7AL, Coventry, UK
[2] Warwick Medical School, University of Warwick, Gibbet Hill Road, CV4 7AL, Coventry, UK
[3] Department of Chemistry, University of Warwick, Gibbet Hill Road, CV4 7AL, Coventry, UK
[4] Institute of Precision Diagnostics and Translational Medicine, Dept of Pathology, UHCW NHS Trust, Clifford Bridge Road, CV2 2DX, Coventry, UK

* Corresponding author email: A.Sagona@warwick.ac.uk

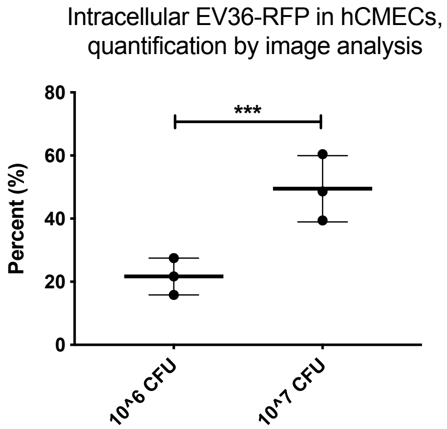

Suppl. Figure 1.

Image quantification of intracellular EV36-RFP of hCMECs. hCMEC cultures were incubated for 1 h with EV36-RFP at concentrations ranging from 10^6 to 10^7 CFU/ml. The cells were fixed and imaged and manual quantification was performed using the ImageJ software.

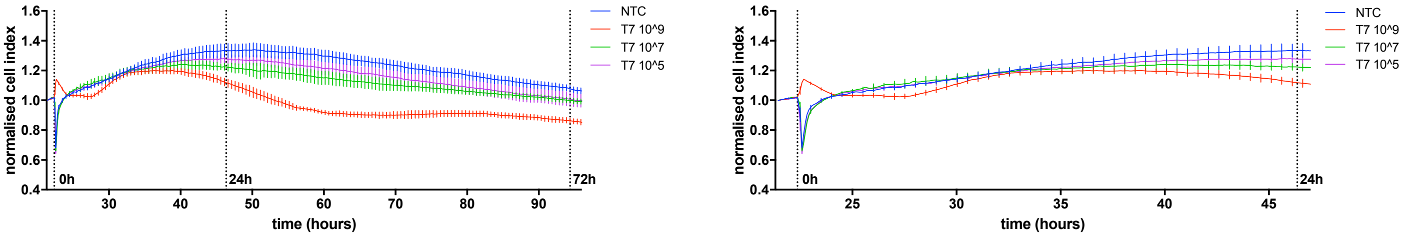

Suppl. Figure 2.

Temporal impedance profiles of hCMEC cultures, as measured using the xCELLigence system, incubated with phage T7 at a concentration range of 10^5 to 10^9 PFU/ml. NTC = Untreated hCMEC cultures. Vertical lines denote the addition of treatment at 0 h, and 24- and 72 h post treatment. The data is presented as the average normalised cell index across the acute (24 hours) and long-term (72 hours) incubate period (+/- SD, n > 3).

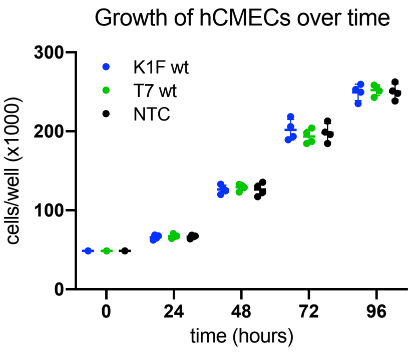

Suppl. Figure 3.

Growth of hCMEC cultures over times. Phages K1F or T7 were added to hCMEC cultures at a final concentration of 10^9 PFU/ml. NTC = untreated bacterial culture. +/- SD, n = 4 in each case.

**Phage preparation**	**Undiluted**	**10^7 PFU/ml**	**10^9 PFU/ml**
Phage K1F-GFP	846 EU/ml	0.086 EU/ml	
Phage K1F	303 EU/ml	0.0060 EU/ml	0.60 EU/ml
Phage T7	523 EU/ml	0.013 EU/ml	1.3 EU/ml
**Media/diluent**			
EndoGRO media	0.15 EU/ml		
DEPC water	0.14 EU/ml		
SM-buffer	< 0.04 EU/ml *		
LB	0.19 EU/ml		
Leibovitz	0.12 EU/ml		

Suppl. Figure 4.
Concentration of endotoxins in phage preparations, media and diluents. Concentrations listed for phage preparations at 10^7 and 10^9 are calculated from stock endotoxin levels based on PFU/ml of undiluted stock. *Detection limit for the LAL Assay used is 0.04 EU/ml.

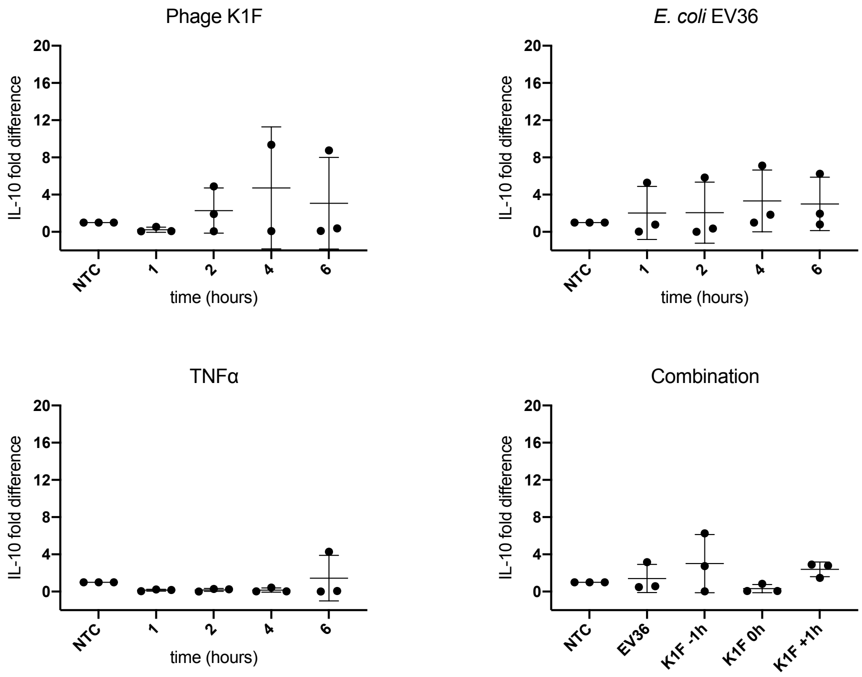

Suppl. Figure 5.
Expression pattern of IL-10 as measured by real-time qPCR. hCMEC cultures were incubated with 10^7 CFU/ml *E. coli* EV36, 10^7 PFU/ml phage K1F or 500 pg/ml TNFα and specific mRNA levels measured over time, or incubated with 10^7 CFU/ml *E. coli* EV36 having 10^4 PFU/ml phage K1F added 1 h before-, simultaneously-, or 1 h after bacterial addition, and incubated for 6 h before RNA harvest. Data were expressed relative to internal control (GAPDH) and then normalised to the untreated control value.
